# Pelubiprofen and Shinbaro: a therapeutic approach for osteoarthritis in a murine medial meniscus destabilization model

**DOI:** 10.3389/fphar.2025.1665393

**Published:** 2025-10-02

**Authors:** Jin Young Hong, Hyunseong Kim, Hyun Kim, Wan-Jin Jeon, Changhwan Yeo, Junseon Lee, Yoon Jae Lee, In-Hyuk Ha

**Affiliations:** Jaseng Spine and Joint Research Institute, Jaseng Medical Foundation, Seoul, Republic of Korea

**Keywords:** osteoarthritis, pelubiprofen, Shinbaro capsules, chondrocytes, medial meniscal instability model

## Abstract

**Introduction:**

Current osteoarthritis treatments can cause serious long-term side effects, and Shinbaro capsules have limited efficacy when used alone. We aimed to determine whether combining pelubiprofen and Shinbaro capsules could offer a more effective and safer treatment strategy for osteoarthritis.

**Methods:**

Primary chondrocytes treated with interleukin-1β were assessed under different conditions: Shinbaro capsules (200 μg/mL), pelubiprofen (25/50 µM), and Shinbaro capsules + pelubiprofen (200 μg/mL and 25 μM, respectively). Male C57BL/6 mice underwent medial meniscus destabilization surgery to induce osteoarthritis and were treated five times a week for 8 weeks with either a vehicle control (saline), Shinbaro capsules alone (100 mg/kg), pelubiprofen alone (1.5/4.5 mg/kg), or Shinbaro capsules + pelubiprofen (100 mg/kg and 1.5 mg/kg, respectively).

**Results:**

Shinbaro capsules + pelubiprofen significantly improved cell viability, inhibited nitric oxide production and lactate dehydrogenase activity, and reduced pro-inflammatory cytokine production more effectively than the individual treatments. Alcian blue staining showed a notable increase in expression intensity with Shinbaro capsules + pelubiprofen. In treated mice, combination treatment significantly reduced the hyaline cartilage/calcified cartilage ratio and synovitis scores, improved the Osteoarthritis Research Society International scores and subchondral bone plate thickness, and enhanced collagen regeneration. Key markers of healthy cartilage and growth plate activity were significantly upregulated, whereas markers involved in cartilage matrix degradation were markedly reduced with Shinbaro capsules + pelubiprofen. Behavioral tests showed significant improvements in pain sensitivity and joint function with combination treatment.

**Discussion:**

Shinbaro capsules + pelubiprofen effectively preserve chondrocytes, reduce inflammation, and alleviate osteoarthritis symptoms through synergistic mechanisms, making it a promising therapeutic strategy for osteoarthritis management.

## Graphical Abstract

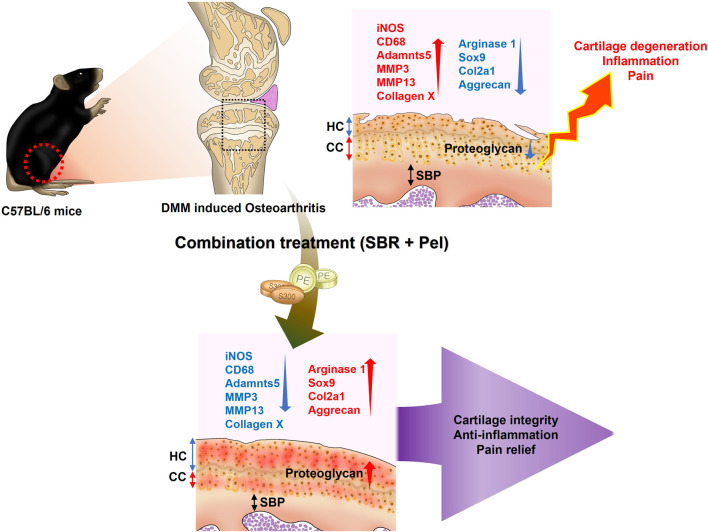

## 1 Introduction

Osteoarthritis (OA) is a common degenerative joint disorder characterized by cartilage breakdown, pain, stiffness, and reduced joint mobility (Drummer et al., 2021). It poses a significant burden on healthcare systems and patients, highlighting the need for effective treatments (Kloppenburg and Berenbaum, 2020). OA management often involves a multifaceted approach, with nonsteroidal anti-inflammatory drugs (NSAIDs) playing a central role in alleviating pain and reducing inflammation (Magni et al., 2021; Gambari et al., 2022). Pelubiprofen (Pel), a specific NSAID, works by inhibiting cyclooxygenase enzymes (COX-1 and COX-2) to reduce inflammation and pain (Choi et al., 2014; Shin et al., 2020). However, long-term NSAID use carries risks such as gastrointestinal ulcers, cardiovascular events, and impaired kidney function, particularly in older individuals or those with pre-existing kidney conditions. This necessitates the exploration of safer alternatives (Marcum and Hanlon, 2010; Bindu et al., 2020).

To address these limitations, capsules of Shinbaro (SBR), a type of natural herbal extract, were developed by Green Cross Corporation, a prominent pharmaceutical company in South Korea (Jung et al., 2019). These capsules were approved by the Korean Ministry of Food and Drug Safety in 2011, marking a significant milestone as they represent an alternative approach to managing conditions such as OA (Ha et al., 2016; Kim et al., 2016). Preclinical studies have demonstrated that SBR (also known as GCSB-5) possesses potent anti-inflammatory and analgesic properties by suppressing key pro-inflammatory mediators, including iNOS, COX-2, TNF-α, and IL-1β. In experimental osteoarthritis models, SBR attenuated proteoglycan loss, reduced extracellular matrix degradation, preserved cartilage integrity, and protected against trabecular bone microarchitecture degeneration. These effects were partly mediated through inhibition of NF-κB activation and reduction of PGE2 production. Notably, no significant toxicity or systemic adverse effects were observed in preclinical studies, supporting its potential safety for therapeutic use (Kim et al., 2016).

Consistent with these preclinical findings, a prospective, randomized, double-blind, multicenter clinical trial in 198 patients with knee osteoarthritis demonstrated that SBR provided clinical efficacy comparable to celecoxib. Improvements in WOMAC scores, pain VAS, and physician’s global assessments were equivalent between groups, while adverse drug reactions tended to occur less frequently in the SBR group, with no severe adverse events reported. Together, these results suggest that SBR offers efficacy equivalent to a selective COX-2 inhibitor while potentially providing a more favorable safety profile (Park et al., 2013). As a natural herbal formulation, SBR may therefore represent a viable long-term therapeutic alternative to synthetic drugs, particularly in older patients or those with comorbidities (Karimi et al., 2015).

Although SBR is generally considered safe, its effectiveness in managing OA symptoms on its own is limited. In response, Korean pharmaceutical companies, with a strong tradition in natural medicines, are developing combination drugs that maximize the synergy between synthetic drugs and bioactive natural pharmaceuticals to enhance efficacy and reduce side effects. This approach is gaining attention in treating cancer, metabolic disorders, and immune diseases, offering new therapeutic options and boosting global market competitiveness (Langeh et al., 2022; Obiang-Obounou and Jang, 2011; Bota et al., 2024; Kim et al., 2015; Cheon and Ko, 2022).

This study hypothesized that a combination therapy of Pel and SBR might provide a more effective and safer approach to managing OA. The study aimed to preserve chondrocytes and reduce OA symptoms using a murine OA model of destabilization of the medial meniscus (DMM) and primary cultured chondrocytes.

## 2 Materials and methods

### 2.1 Preparation of SBR and pel tablets

SBR capsules (300 mg/tablet) were purchased from GC Biopharma Corp. (Yongin, Korea), and Pel capsules (30 mg/tablet) were obtained from Daewon Pharmaceuticals Co., Ltd. (Seoul, Korea) for use in both *in vitro* and *in vivo* experiments. Each SBR tablet (300 mg) contains dried extracts (20→1) of six oriental herbs: *Acanthopanax* sessiliflorus, *Achyranthis* radix, *Saposhnikoviae* radix, *Eucommiae* cortex, *Cibotii* rhizoma, and *Glycine semen* nigra, according to the information provided by the Korea Pharmaceutical Information Center.

### 2.2 Primary culture of mice chondrocytes

Primary chondrocytes were cultured using cartilage isolated from 4–5-day-old pups (Orient Bio, Gyeonggi-do, South Korea; approval number JSR-2024-03-005-A, Jaseng Animal Care and Use Committee) according to an existing culture method (Cheon and Ko, 2022). Briefly, 4–5-day-old pups were euthanized using 2%–3% isoflurane gas (Forane; BK Pharm, Goyang, Republic of Korea). After sacrifice, the mice’ legs were bent, skinned, and the knees dissected using curved scissors. The tissues were collected into a 50 mL tube containing 1% penicillin-streptomycin (P/S, 10,000 U/mL; Thermo Fisher Scientific Inc., Waltham, MA, USA) with phosphate-buffered saline (PBS; Welgene, Gyeongsan, Republic of Korea). Tissues were initially washed with vigorous shaking, followed by pipetting and three washes. They were then treated with serum-free Dulbecco modified Eagle medium (DMEM; Hyclone, Logan, UT), supplemented with 0.2% collagenase type II (Worthington, Lakewood, NJ, USA) and 0.1% trypsin (Thermo Fisher Scientific Inc.), and incubated at 37 °C for 3 h. After incubation, the tissues were tapped vigorously for dissociation; if not fully dissociated, they were incubated for an additional 10–20 min. Contaminating tissues were removed using forceps, leaving only the white, heart-shaped cartilage, which was washed again in a new tube. A second treatment was conducted with serum-free DMEM containing 0.2% collagenase type II, followed by a 2 h incubation at 37 °C. The tissues were tapped again after 2 h and pipetted to ensure complete dissociation, with additional incubation if necessary. Once fully disconnected, the cartilage was suspended in DMEM containing 10% fetal bovine serum (FBS; Thermo Fisher Scientific Inc.) to stop the enzymatic reaction. The suspension was filtered through a 40-µm cell strainer (VWR, Radnor, PA, USA) to remove debris and centrifuged at 1,200 rpm for 3 min to collect the cell pellet. The pellet was resuspended in high-glucose DMEM containing 10% FBS and 1% P/S. The cells were then seeded into 96- or 24-well culture dishes and cultured for 3 days.

### 2.3 *In vitro* drug treatment

Primary chondrocytes were cultured for 3 days before drug treatment. SBR was administered alone at various concentrations (0, 25, 50, 100, 200, 400, and 800 μg/mL) or in combination with 10-ng/mL interleukin (IL)-1β (Peprotehch, Cranbury, NJ, USA). Similarly, Pel was administered alone at various concentrations (0, 1, 10, 25, 50, 100, and 200 μM) or concurrently with 10-ng/mL IL-1β. To assess the effectiveness of the combination therapy with SBR and low-dose Pel (25 μM, referred to as Pel-25) compared to high-dose Pel monotherapy (50 μM, referred to as Pel-50), the Pel-50 group was included. Consequently, six groups were evaluated: a non-treatment group (Blank), an IL-1β treatment group (10-ng/mL IL-1β), the SBR-200 group (200-µg/mL SBR+10-ng/mL IL-1β), the Pel-25 group (25-µM Pel+10-ng/mL IL-1β), the Pel-50 group (50-µM Pel+10-ng/mL IL-1β), and the SBR + Pel group (200-µg/mL SBR+25-µM Pel+10-ng/mL IL-1β).

### 2.4 Cell viability/cytotoxicity assays

Cell viability was assessed using a Cell Counting Kit-8 assay (CCK-8; Dojindo, Kumamoto, Japan) following the method described by Hong et al. (2024). Mice chondrocytes were seeded in 96-well plates at 8 × 10^3^ cells/100 µL and treated with varying concentrations of SBR or Pel with or without IL-1β. After a 48-h incubation period, CCK-8 solution (10% of the total volume) was added to each well and incubated for an additional 4 h. Absorbance was measured at 450 nm using a BioTek Epoch Microplate Reader (BioTek Instruments Inc., Winooski, VT, USA) to determine cell viability. Mice chondrocytes were seeded at a density of 8 × 10^3^ cells per 100 μL in a 96-well plate, and drug treatments were administered under six different conditions (Blank, IL-1β, SBR-200, Pel-25, Pel-50, SBR + Pel groups). A nitric oxide (NO) assay was performed to quantify NO production. After 48 h of drug treatment under IL-1β insult, 50 µL of cell culture medium was mixed with 50 µL of Griess reagent (2% sulfanilamide in 5% phosphoric acid; Sigma-Aldrich, St. Louis, MO, USA) at a 1:1 ratio (v/v) to measure NO production. Fresh culture medium served as a blank for all experiments. Nitrite quantity was determined using a sodium nitrite standard curve. A lactate dehydrogenase (LDH) assay (Abcam, Cambridge, UK) was performed according to the manufacturer’s guidelines. After 48 h of drug treatment, cells were collected from the 96-well culture plates, and the assay reaction mixture was added to each well. Optical density was measured at 450 nm using an Epoch microplate reader, with readings taken every 2 min for 1 h to monitor LDH release.

### 2.5 Alcian blue staining

Chondrocytes at a concentration of 1 × 10^6^ cells/mL were seeded in 40-µL aliquots into a 24-well plate containing DMEM, high-glucose medium with 10% FBS, and 1% P/S. After 4 h of incubation, 500 µL of culture medium was added to each well, and the cells were cultured for 3 days. Next, the cells were treated with SBR (200 μg/mL), two concentrations of Pel (25 and 50 µM), or a combination of SBR and Pel, either with IL-1β or in a control group treated only with IL-1β, and cultured for 48 h. Subsequently, the cells were fixed with 4% paraformaldehyde (PFA, Biosesang, Seongnam, Republic of Korea). The cells were then stained with 1% Alcian blue 8GX (Sigma-Aldrich) in 0.1N HCl at room temperature for 2 h and washed with PBS. Images of the stained sections were captured at ×10 magnification using an inverted microscope (Nikon, Tokyo, Japan). Alcian blue intensity was quantified using ImageJ software (National Institutes of Health, Bethesda, MD, USA). The process involved using ImageJ: selecting Image - Color - Color deconvolution and then choosing the Alcian blue channel to isolate the blue areas. Measurements were taken after setting the threshold value.

### 2.6 Enzyme-linked immunosorbent assay (ELISA)

IL-6 and IL-1β levels were analyzed using ELISA kits. The culture media were collected 48 h after drug treatment from mice chondrocytes cultured at a density of 4 × 10^4^ cells/500 µL in a 24-well plate. The IL-6 and IL-1β levels were then measured using ELISA kits (ThermoFisher Scientific, Inc.) according to the manufacturer’s instructions, with absorbance measured at 450 nm using a BioTek Epoch Microplate Reader.

### 2.7 Immunocytochemistry

Chondrocytes from mice, prepared at a density of 4 × 10^4^ cells/500 µL in a 24-well plate, were treated with the drugs for 48 h according to the group conditions. After treatment, the cells were fixed with 4% PFA (Biosesang) for 30 min, followed by three washes with PBS for 5 min each. After removing PBS, the cells were treated with 0.2% Triton X-100 in PBS for 5 min, followed by two additional PBS washes. The cells were then incubated with 2% normal goat serum (NGS) in PBS at room temperature for 1 h. The primary antibodies used were iNOS (1:200; ThermoFisher), MMP3 (1:50; Abcam), and MMP13 (1:100; Abcam). These antibodies were applied and incubated overnight at 4 °C. Following three washes with PBS, the secondary antibodies—FITC-conjugated goat anti-rabbit IgG (1:300; Jackson Immuno-Research Labs, West Grove, PA, USA) and Rhodamine-conjugated goat anti-rabbit IgG (1:300; Jackson Immuno-Research Labs)—were applied at room temperature for 2 h. Subsequently, the cells were washed three times with PBS and stained with 4′,6-diamidino-2-phenylindole (DAPI, 1:1,000; Tokyo Chemical Industry Co., Tokyo, Japan) for 10 min. Another PBS wash was performed before mounting the cells with a fluorescence mounting medium (Dako; Agilent, Santa Clara, CA, USA). Imaging was conducted using a confocal microscope (Eclipse C2 Plus; Minato, Tokyo, Japan), and fluorescence intensity was quantified using ImageJ software.

### 2.8 DMM surgery-induced OA models

DMM surgery on the right knee was performed on 8-week-old male C57BL/6 mice (Samtako Bio, Gyeonggi, Korea) with approval from the Jaseng Animal Care and Use Committee (approval number: JSR-2024-01-001-A). Mice were anesthetized with 2%–3% isoflurane gas (Forane; BK Pharm) and placed in the supine position on the surgical table. The fur on the right knee was shaved, and the joint capsule medial to the patellar tendon was incised with scissors. The medial meniscotibial ligament was carefully transected without damaging the cruciate ligaments. In the sham operation, the medial meniscotibial ligament was exposed in the same manner but not transected. Thus, the sham group underwent all surgical procedures (anesthesia, incision, joint exposure, and closure) except for the critical injurious step of ligament transection. This approach is consistent with the standard sham surgery described by Glasson et al. where the ligament is visualized but not cut (Glasson et al., 2007). The joint capsule and skin were sutured using black silk (5/0; Ailee Co., Ltd., Busan, Korea). A total of 10 animals per group were subjected to DMM surgery, and all animals successfully underwent induction, resulting in a success rate of 100%. Postoperative pain and distress were monitored daily in accordance with animal ethical guidelines. Humane endpoints were predefined as >20% body weight loss, persistent abnormal appearance or posture, severe immobility not relieved by supportive care, or moribund condition. Environmental enrichment was provided to minimize stress.

### 2.9 *In vivo* drug administration

Drug treatments began 1 week after DMM surgery and were administered orally five times a week for 8 weeks. The study included six groups: a sham group and a DMM control group, both receiving saline; an SBR group receiving 100 mg/kg of SBR; Pel-1.5 and Pel-4.5 groups receiving 1.5 mg/kg and 4.5 mg/kg of Pel, respectively; and an SBR-Pel group receiving 100 mg/kg SBR and 1.5 mg/kg Pel. After the treatment period, the animals were euthanized. Euthanasia was conducted following the AVMA Guidelines for the Euthanasia of Animals (2020 Edition) using CO_2_ inhalation at a fill rate of 30% chamber volume/min, followed by cervical dislocation to ensure rapid and humane death.

### 2.10 Safranin-O/fast green staining

After perfusing the mice with 0.9% saline (Sigma-Aldrich) via cardiac infusion, the right knee was excised. The knee was then immersed in 4% PFA (Biosesang) for 2 days for fixation. After fixation, the tissues were embedded in a paraffin block. The block was secured in a HistoCore MULTICUT microtome (LEICA, Wetzlar, DE) and sectioned to a thickness of 5 µm. Next, the tissue sections were floated in water at 40 °C for approximately 2–3 min to flatten them, before being mounted onto coated slides. Finally, the slides were dried on a hotplate set to 37 °C. Safranin-O/Fast Green staining (Sigma-Aldrich) was performed according to the manufacturer’s instructions. Stained sections were imaged using an inverted microscope (Eclipse C2 Plus; Nikon). The Osteoarthritis Research Society International (OARSI) grade was assessed according to the methodology described in previous studies (Glasson et al., 2010). Additionally, the subchondral bone plate (SBP) thickness was measured using images captured at ×40 magnification. These measurements were conducted using ImageJ software, as outlined in previously reported protocols (Nagira et al., 2020).

### 2.11 Hematoxylin and eosin (H&E) staining

H&E staining was performed on paraffin-embedded tissues to calculate the hyaline cartilage (HC)/calcified cartilage (CC) ratio and synovitis score. Sections were immersed in hematoxylin for 2 min and 30 s, rinsed in running tap water for 2 min, and stained with eosin for 50 s. They were then dehydrated using a graded ethanol series (70%–100%) and cleared with xylene. Stained sections were imaged using a light microscope (Nikon) at ×100 magnification. The HC/CC ratio was calculated by measuring HC and CC thicknesses with ImageJ software (National Institutes of Health). Synovitis was assessed by evaluating changes in the synovial lining thickness and cellular density in the synovial stroma using a standardized synovitis scoring table (0–3 points per parameter, with a maximum possible score of 6 points) (Nekomoto et al., 2023).

### 2.12 Masson trichrome staining

Masson trichrome staining was performed on paraffin-embedded knee joint tissues using a Trichrome Stain (Masson) Kit (Abcam). The collagen fibers were stained blue, whereas the background appeared red. After staining, the images were captured using a Nikon light microscope. The area and pixel density of the blue-stained regions, representing the collagen content and distribution, were quantified for each group using ImageJ software.

### 2.13 Immunohistochemistry (IHC)

For IHC, the slides were first deparaffinized by placing them in a dry oven at 60 °C for 1 h to melt the paraffin, followed by soaking in xylene twice for 10 min each. They were then soaked in a 1:1 mixture of xylene and 100% ethanol for 3 min and sequentially rehydrated in 100%, 90%, 80%, and 70% ethanol for 2 min each. Slides were washed in distilled water and PBS for 5 min each. Antigen retrieval was performed by heating the slides in citrate buffer at 100 °C for 20 min, followed by incubation in citrate buffer at room temperature for an additional 20 min. The slides were placed in a humid chamber and permeabilized with a 0.5% Triton X-100 solution for 30 min. Blocking was performed by incubating the slides in a 2% NGS solution for 1 h without washing. Primary antibodies (SOX9 [1:500; Abcam], collagen 2 [1:100; Invitrogen, Waltham, MA, USA], aggrecan [1:500; Proteintech, IL, USA], collagen X [1:200; Abcam], Adamts5 [1:100; Invitrogen], matrix metallopeptidase [MMP]-3 [1:50; Abcam], MMP13 [1:200; Abcam], and PCNA [1:100, Saint Johnson Lab, London, UK]) were applied and incubated overnight at 4 °C. Then, the slides were washed twice with PBS and incubated with secondary antibodies (FITC-conjugated goat anti-rabbit IgG [1:300; Jackson Immuno-Research Labs, West Grove, PA, USA], rhodamine-conjugated goat anti-rabbit IgG [1:300; Jackson Immuno-Research Labs], and goat anti-mouse IgG antibody [H + L]) for 2 h at room temperature. Next, the slides were washed with PBS and treated with DAPI (1:1,000; Tokyo Chemical Industry Co.) for 10 min to stain the nuclei, washed again, and mounted with a suitable mounting medium (Dako). For PCNA detection, the slides were subjected to diaminobenzidine staining (DAB; Vector Laboratories, Burlingame, CA, USA). After treatment with the primary PCNA antibody, they were incubated with a biotinylated secondary antibody (1:400; Vector Laboratories). Following a PBS wash, the slides were treated with an ABC kit (Vector Laboratories) for 20 min. After another PBS wash, the slides were incubated with DAB solution (Vector Laboratories) for 2 min and 30 s. The slides were subsequently rinsed and washed thoroughly with PBS, counterstained with hematoxylin for 20 s, and washed with distilled water. Finally, the slides were dehydrated through a graded alcohol series, immersed in xylene for 2 min, and mounted. Images were generated using Z-stack and tile scan projections at 100× or ×400 magnification, and signal intensities were quantified using ImageJ software.

### 2.14 Body weight measurements

The body weight of each mouse was measured every 7 days for 8 weeks using an electronic scale (Sartorius, Gottingen, Germany). For each mouse, body weight gain as a percentage of the initial weight was calculated using the following formula:

$$\text{Percentage of body weight gain }\left(\%\right)=\left(\frac{\;Final\;weight,8\;weeks-Initial\;weight,0\;week}{Initial\;weight,0\;week}\right)\times 100$$

### 2.15 Blood chemistry analysis

To confirm the *in vivo* safety of the drug, blood biochemical analysis was conducted on serum samples. Blood samples were collected from the retroorbital plexus under anesthesia and centrifuged at 3,000 *g* for 10 min at room temperature. The serum was then carefully transferred to new 1.5-mL tubes and stored at −80 °C until analysis. The serum was later tested using a blood chemistry analyzer (DRI-CHEM NX600; Fujifilm, Tokyo, Japan) to evaluate various biochemical markers.

### 2.16 Weight-bearing test

In OA models, unilateral pain, especially joint pain, is traditionally assessed by measuring weight distribution between the hind paws, which indicates the degree of joint pain. Measurements were taken five times: before OA induction and at 2, 4, 6, and 8 weeks post-induction. The Incapacitance tester (Ugo Basile, 47885) was used to position the mice in an acrylic frame, ensuring both hind paws were on the weight measurement devices. The average weight distribution on both hind paws was measured three times per animal within 3 s. The weight-bearing ratio between the left and right paws was calculated, and the average ratio was recorded as the final weight-bearing rate. Additionally, the improvement rate in weight-bearing due to pain was calculated using the following formula.

$$\text{Improvement rate of weight}-\text{bearing }\left(\%\right)=\left(\frac{\;\text{Average}\;\text{weight}-\text{bearing}\;\text{of}\;\text{the}\;\text{experiment}\;\text{group}\;\text{at}\;8\;\text{weeks}-\text{Average}\;\text{weight}-\text{bearing}\;\text{of}\;\text{the}\;\text{DMM}\;\text{group}\;\text{at}\;8\;\text{weekss}}{\text{Average}\;\text{weight}-\text{bearing}\;\text{of}\;\text{the}\;\text{DMM}\;\text{group}\;\text{at}\;8\;\text{weeks}}\right)\times 100$$

### 2.17 Von frey test

The Von Frey test assessed mechanical nociception sensitivity by contacting the plantar skin of the paw with a Von Frey filament to measure the minimum force required for a withdrawal response and the latency of this reaction. These metrics indicate the sensitivity of an animal to mechanical pain. Measurements were taken at five points: before OA induction and at 2, 4, 6, and 8 weeks after drug administration. Using the Von Frey apparatus (Ugo Basile, 38450), mice were placed in an acrylic frame designed for Von Frey testing and allowed to stabilize for 30 min. The filament was positioned on the plantar surface of the mouse, and the latency until paw withdrawal was recorded. Each mouse was tested five times, and the average latency time was calculated. The final rate of improvement in mechanical pain sensitivity was calculated using the following formula.

$$\text{Improvement rate of mechanical pain }\left(\%\right)=\left(\frac{\;Average\;latency\;of\;the\;experiment\;group\;at\;8\;weeks-Average\;latency\;of\;the\;DMM\;group\;at\;8\;weeks}{Average\;l\text{atency}\;of\;the\;DMM\;group\;at\;8\;weeks}\right)\times 100$$

### 2.18 Statistical analysis

Data are presented as mean ± standard deviation. Group comparisons were performed using one-way analysis of variance, followed by the Tukey post-hoc analysis using GraphPad Prism software (GraphPad, Inc., La Jolla, CA, USA). Statistical significance was set at P < 0.05.

## 3 Results

### 3.1 Synergistic inhibition of cytotoxicity and enhancement of anti-inflammatory responses by SBR and pel combination treatment in IL-1β-induced primary mice chondrocytes

**FIGURE 1:**
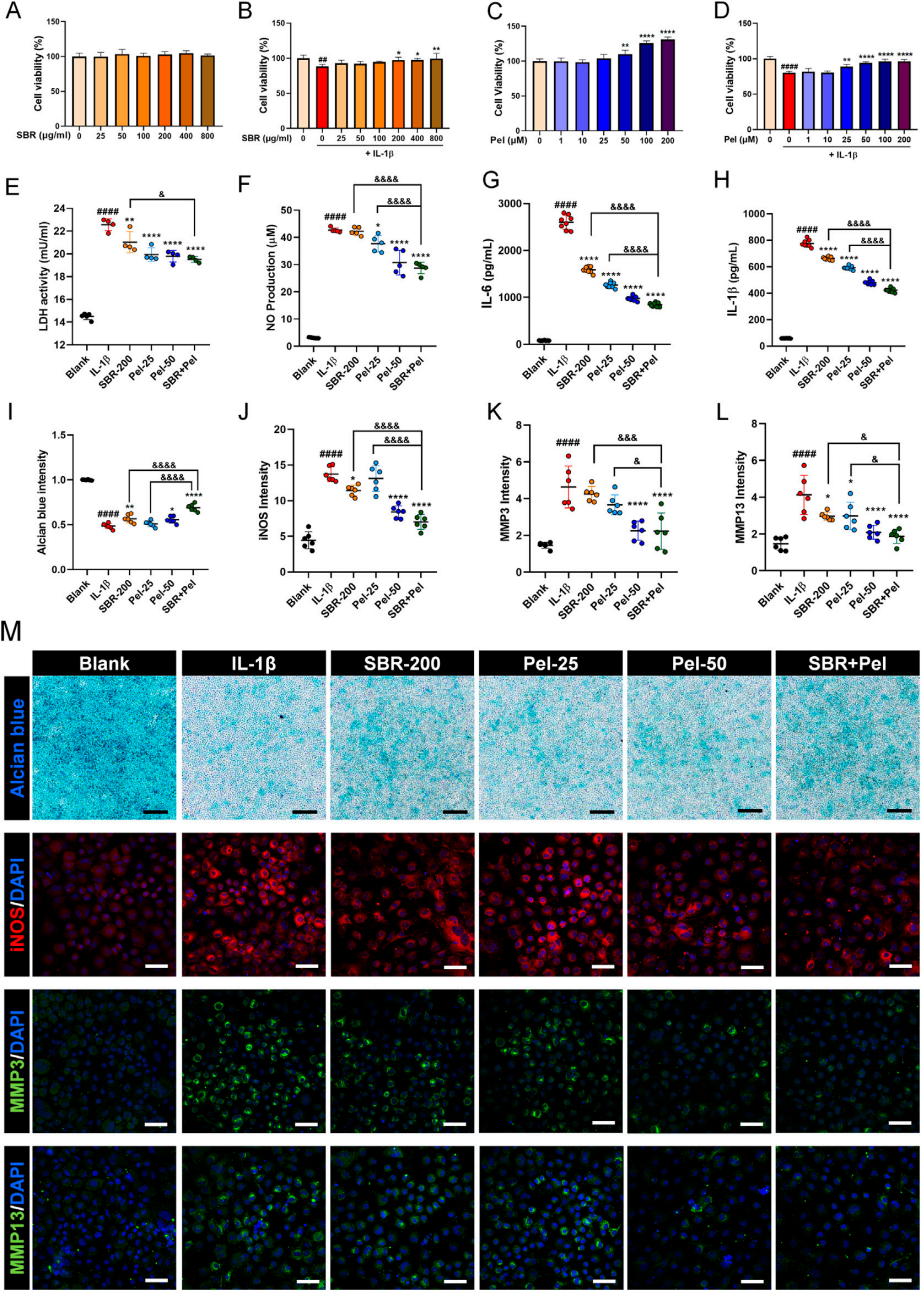
*In vitro* assays evaluating the synergistic effects of on cell viability, cytotoxicity, inflammatory markers, and proteoglycan accumulation in IL-1β-treated primary mice chondrocytes. **(A,B)** Graphs showing cell viability of primary chondrocytes treated with varying concentrations of SBR (0–800 μg/mL) for 48 h, with or without IL-1β (10 ng/mL), assessed by CCK-8 assay (n = 6). **(C,D)** Graphs displaying cell viability of primary chondrocytes treated with varying concentrations of Pel (0–200 μM) for 48 h, with or without IL-1β (10 ng/mL), assessed by CCK-8 assay (n = 6). **(E,F)** Cytotoxicity assays measuring LDH activity and NP production in the Blank, IL-1β, SBR-200, Pel-25, Pel-50, and SBR + Pel groups. **(G,H)** ELISA analyses measuring the levels of pro-inflammatory cytokines IL-6 and IL-1β in the Blank, IL-1β, SBR-200, Pel-25, Pel-50, and SBR + Pel groups. **(I)** Quantitative analysis of Alcian blue staining intensity in each group. **(J–L)** Quantitative fluorescence intensity of iNOS, MMP3, and MMP13 from immunocytochemically stained images in each group. **(M)** Representative images of Alcian blue staining and iNOS, MMP3, and MMP13 immunocytochemical staining in each group. Black scale bar = 100 μm, white scale bar = 50 μm. Data are expressed as the mean ± standard deviation. Significant differences were analyzed via one-way analysis of variance with Tukey’s post-hoc analysis as follows: ^##^p < 0.01 and ^####^p < 0.0001 compared with blank group; ^*^p < 0.05, ^**^p < 0.01, and ^****^p < 0.0001 compared with IL-1β group; ^&^p < 0.05, ^&&&^p < 0.001, and ^&&&&^p < 0.0001 compared with SBR-200 or Pel-25 groups.

SBR showed no cytotoxicity up to 800 μg/mL (Figure 1A). Co-treatment with IL-1β at this concentration range significantly increased cell viability starting from 200 μg/mL of SBR (Figure 1B). For Pel, a significant increase in cell viability compared to the blank was observed starting at 50 µM (Figure 1C). When co-treated with IL-1β, cell viability significantly increased from 25 µM compared to the IL-1β group (Figure 1D). Consequently, the optimal concentration for the combination therapy was determined to be 200 μg/mL SBR+25 µM Pel.

To evaluate the synergistic inhibitory effect of the combination of SBR and Pel on cytotoxicity in IL-1β-induced primary mice chondrocytes, LDH and NO assays were performed. LDH activity significantly increased in the IL-1β group compared with the blank group. However, it was markedly decreased in the SBR-200, Pel-25, and Pel-50 groups. Notably, the combination treatment of SBR and Pel not only significantly reduced LDH activity compared with the IL-1β group but also exhibited a more pronounced reduction than the SBR-alone group, achieving an inhibitory effect similar to that of higher doses of Pel alone (Pel-50) (Figure 1E). IL-1β treatment resulted in a significant increase of NO compared with the blank. However, the Pel-25, Pel-50, and SBR + Pel treatments significantly reduced NO production compared with the IL-1β group. SBR + Pel exhibited a notable synergistic effect, achieving NO reduction levels comparable to those seen with high-concentration Pel (Pel-50) treatment and significantly more than those with the SBR-200 and Pel-25 treatments alone (Figure 1F).

ELISA analyses revealed that the levels of pro-inflammatory cytokines IL-6 and IL-1β significantly increased upon IL-1β treatment. However, treatment with the SBR-200, Pel-25 or Pel-50, and SBR + Pel significantly decreased these cytokine levels compared with the IL-1β group. SBR + Pel exhibited a more significant anti-inflammatory effect than either SBR-200 or Pel-25 alone (Figures 1G,H). Alcian blue staining, specific for chondrocytes, indicated proteoglycan accumulation. Staining intensity was measured and compared among the groups. IL-1β treatment significantly reduced intensity compared to the blank, while SBR, Pel-50, and SBR + Pel treatments significantly increased intensity compared to IL-1β. Notably, SBR + Pel exhibited a synergistic effect, showing a higher intensity than SBR or Pel-25 alone, with mean intensity levels surpassing those of the Pel-50 (Figures 1I,M). ICC results showed that iNOS and MMP3 expression increased 3-fold with IL-1β but was reduced by more than half in the SBR + Pel group, which also had the lowest MMP13 expression levels, indicating a significant reduction compared to IL-1β and highlighting the combination’s efficacy (Figures 1J–M).

### 3.2 Histological analysis of the synergistic benefits of SBR and pel combination therapy on enhanced cartilage preservation and OA mitigation

**FIGURE 2:**
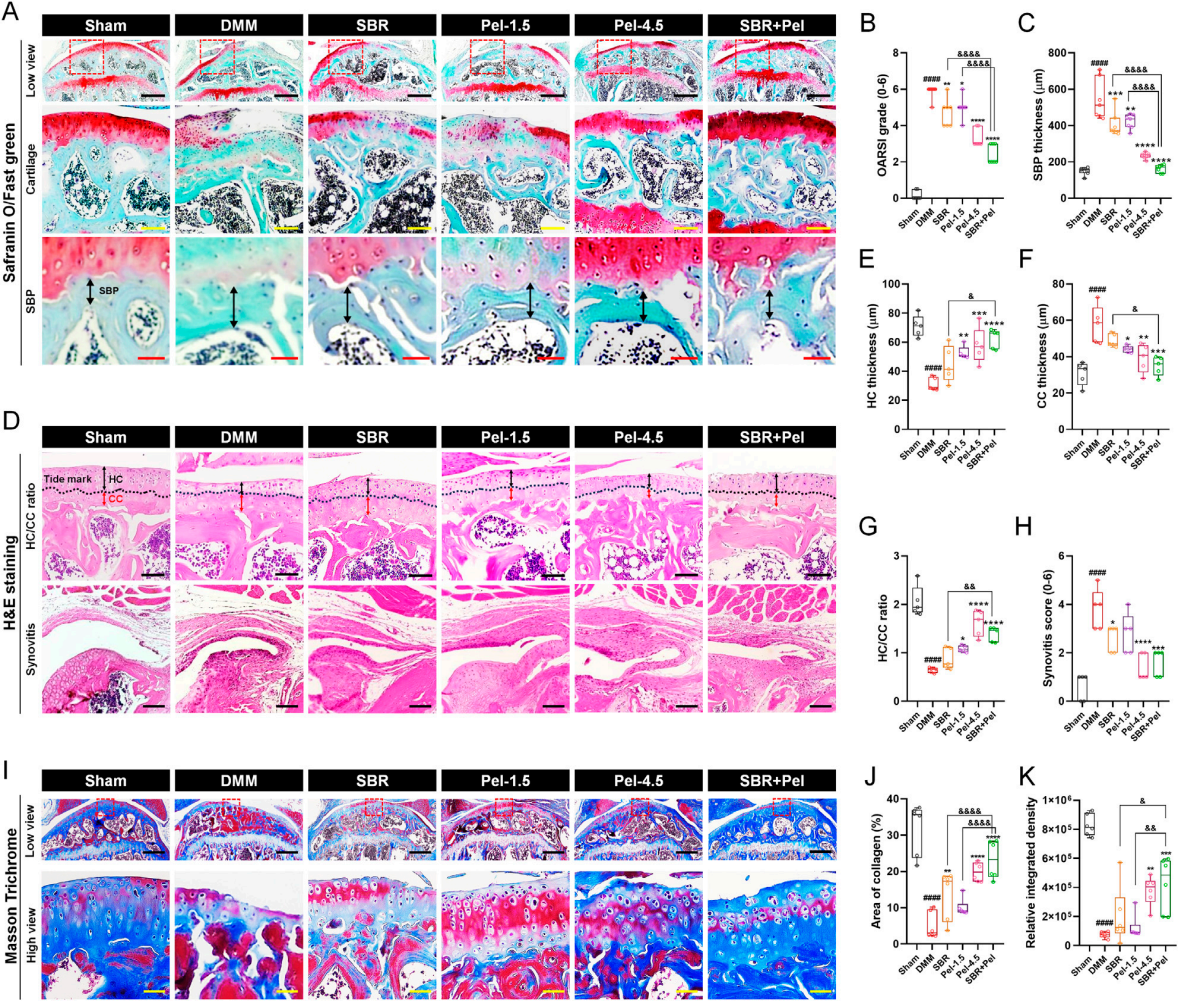
Histological staining analysis of cartilage integrity and OA progression in joint tissues obtained at 8 weeks after DMM-induced OA, with SBR and Pel administrations. **(A)** Representative images of Safranin-O/Fast Green staining in joint tissues from Sham mice, DMM-operated mice, and DMM-operated mice treated with each drug. Safranin-O stains proteoglycan-rich cartilage matrix red/orange, whereas Fast Green stains the background and non-collagenous tissue blue/green. The dashed red squares in the low view images indicate the regions that are shown at higher magnification in the lower panels. Black scale bar = 400 μm, yellow scale bar = 80 μm, and red scale bar = 30 μm. **(B)** Graph assessing the Osteoarthritis Research Society International (OARSI) grade (0-6) to evaluate cartilage integrity and OA progression from Safranin-O/Fast Green-stained images in each group. **(C)** Subchondral bone plate (SBP) thickness measured from Safranin-O/Fast Green-stained images in each group. **(D)** Representative images of H&E-stained articular cartilage for each group. H&E staining was used to distinguish hyaline cartilage (HC) and calcified cartilage (CC) layers. Black scale bar = 100 μm. **(E–G)** Quantitative measurements of HC thickness, CC thickness, and HC/CC ratio from H&E-stained images in each group. **(H)** Synovitis score determined from H&E-stained synovial tissues in each group. **(I)** Representative images of Masson’s trichrome-stained articular cartilage to determine collagen content in each group. Collagen fibers are stained blue, cytoplasm and muscle appear red, and nuclei are stained dark purple. Black scale bar = 400 μm and yellow scale bar = 50 μm. **(J,K)** Quantification of the area and integrated density of blue-stained collagen in each group. Data are expressed as the mean ± standard deviation. Significant differences were analyzed via one-way analysis of variance with Tukey’s post-hoc analysis as follows: ####p < 0.0001 compared with Sham group; *p < 0.05, **p < 0.01, ***p < 0.001, and ****p < 0.0001 compared with DMM group; &p < 0.05, &&p < 0.01, and &&&&p < 0.0001 compared with SBR or Pel-1.5 groups.

On Safranin-O/Fast Green staining (Figure 2A), we observed an increase in the OARSI grade (score 5.9) and SBP thickness (541.7 µm) in the DMM-induced OA group compared with the sham group. SBR and Pel-1.5 treatment significantly reduced these parameters (OARSI scores of 4.7 and 5.0, SBP thickness of 406.4 µm and 421.4 µm, respectively), with the SBR + Pel combination resulting in a more than 2-fold greater reduction than either treatment alone, aligning closely with the sham group results for SBP thickness (Figures 2B,C).

On H&E staining (Figure 2D), the DMM model showed a decrease in HC thickness, an increase in CC thickness, and a threefold reduction in the HC/CC ratio (to 0.6). Additionally, the synovitis score (3.8) was significantly higher than that of the sham group. Administration of SBR and Pel-1.5 alone increased HC thickness, decreased CC thickness, and improved HC/CC ratio. The combination of SBR and Pel demonstrated a synergistic effect on the HC/CC ratio, yielding results similar to those observed for the Pel-4.5 group. Synovitis scores significantly decreased in the SBR and Pel-4.5 groups, while the SBR + Pel treatment led to substantial improvement compared to the Pel-4.5. However, there were no significant differences between the SBR + Pel treatment and either SBR or Pel-1.5 treatments (Figures 2E–H).

Collagen was quantified using Masson’s trichrome staining, which highlighted the collagen fibers in blue (Figure 2I). Both the collagen-positive area and density were significantly lower in the DMM-induced OA group, by 6-fold and 7-fold respectively, compared to the sham group. Conversely, the collagen-positive area showed a significant increase in the SBR, Pel-4.5, and SBR + Pel groups compared with the DMM group. Specifically, SBR + Pel resulted in a further increase in collagen-positive areas compared with either SBR or Pel-1.5 alone, and it achieved a higher overall collagen distribution than the Pel-4.5 treatment (Figures 2J,K).

### 3.3 Synergistic effects of combined SBR and pel therapy on inflammation and cartilage marker expression in OA-induced knee joints

**FIGURE 3:**
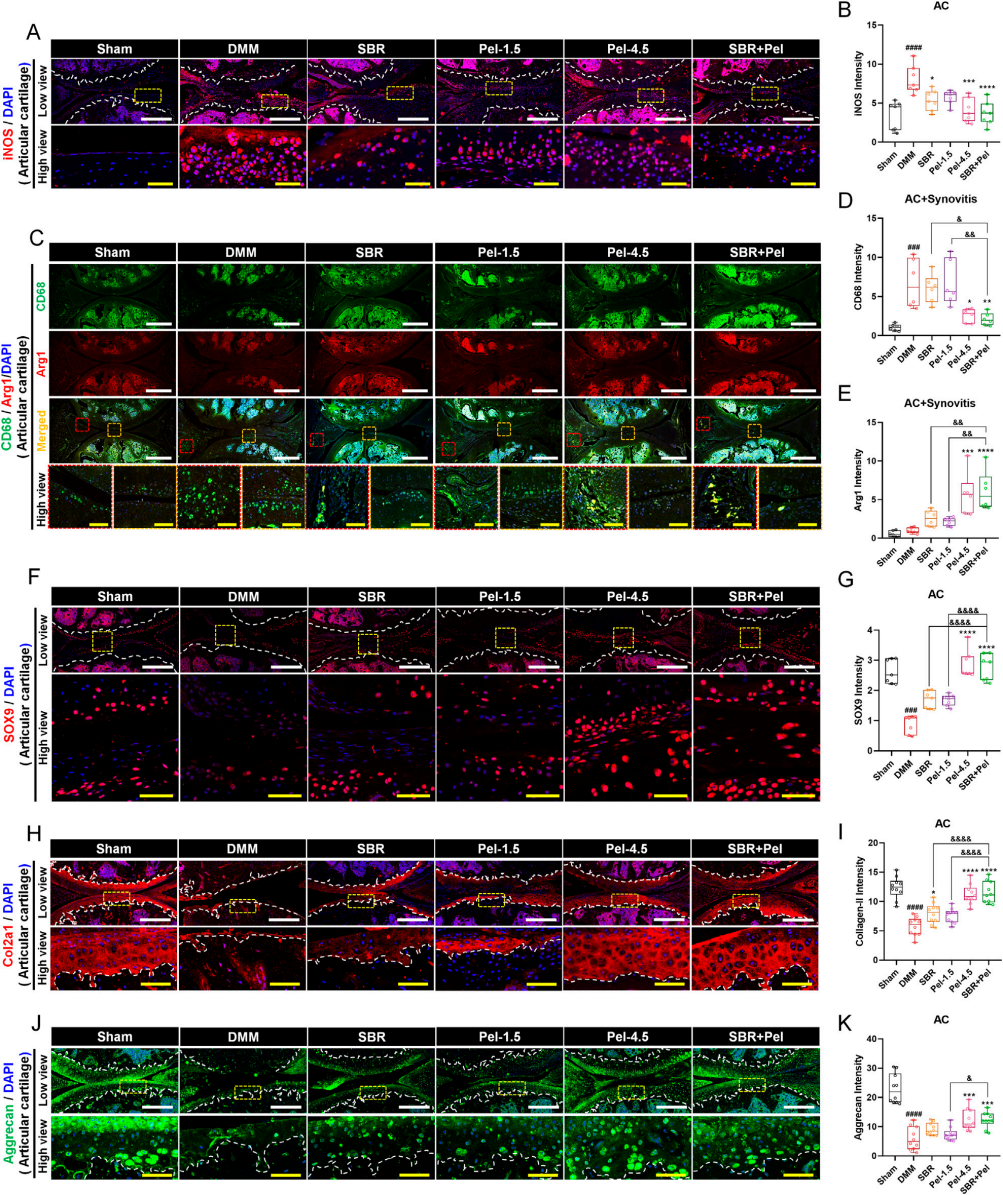
Immunohistological staining analysis of inflammation and cartilage biomarkers in joint tissues obtained at 8 weeks after DMM-induced OA, with SBR and Pel administrations. **(A,B)** Representative fluorescence images of iNOS (red) in each group, along with a quantification graph measuring expression intensity. White scale bar = 200 μm, yellow scale bar = 50 μm. **(C–E)** Representative fluorescence images showing CD68 (green) and Arg1 (red) in each group, along with a quantification graph measuring the intensity of each marker. White scale bar = 500 μm, yellow scale bar = 75 μm. **(F,G)** Representative fluorescence images of SOX9 (red) in cartilage of each group, along with a quantification graph measuring expression intensity. White scale bar = 200 μm, yellow scale bar = 50 μm. **(H,I)** Representative fluorescence images of Col2a1 (red) in cartilage of each group, along with a quantification graph measuring expression intensity. White scale bar = 200 μm, yellow scale bar = 50 μm. **(J,K)** Representative fluorescence images of aggrecan (green) in cartilage of each group, along with a quantification graph measuring expression intensity. White scale bar = 200 μm, yellow scale bar = 50 μm. White dashed lines indicate the boundary of the articular cartilage, and red dashed squares indicate synovitis regions in the joint tissue, and yellow dashed squares indicate cartilage regions at higher magnification in the lower panels. DAPI (blue) was used as a nuclear counterstain in all fluorescence images. Abbreviations: AC, Articular Cartilage. Data are expressed as the mean ± standard deviation. Significant differences were analyzed via one-way analysis of variance with Tukey’s post-hoc analysis as follows: ^####^p < 0.0001 compared with Sham group; ^*^p < 0.05, ^**^p < 0.01, ^***^p < 0.001, and ^****^p < 0.0001 compared with DMM group; ^&^p < 0.05, ^&&^p < 0.01, and ^&&&&^p < 0.0001 compared with SBR or Pel-1.5 groups.

We conducted IHC analysis to evaluate inflammatory response biomarkers, including iNOS, CD68, and Arginase1, as well as key markers related to cartilage formation and maintenance, including SOX9, Collagen type 2 (Col2a1), and aggrecan. This analysis provided crucial insights into the physiological and pathological state of knee joint cartilage under the new combination therapy of SBR and Pel. The expression of iNOS and CD68 markers was minimal in the sham group but increased significantly after DMM induction, primarily in cartilage and synovitis. The M2 macrophage marker arginase 1 showed low expression (Figures 3A–D). The SBR and Pel-1.5 groups exhibited reduced iNOS expression, with the SBR group showing a significant decrease, although CD68-positive macrophages remained similar to the DMM group. In the Pel-4.5 and SBR + Pel groups, both iNOS and CD68 expression levels were significantly reduced compared to the DMM group. Most CD68 macrophages in these groups expressed arginase 1, with significantly increased arginase 1 expression compared to DMM and individual groups (Figures 3C–E). SOX9, a transcription factor known to play a crucial role in chondrocyte differentiation and maintenance, was barely expressed in the cartilage regions induced by DMM. The expression intensity of SOX9 in knee joint cartilage was significantly reduced in the DMM group compared to the sham group. In the SBR or Pel-1.5 groups, SOX9 expression showed an average increase compared to the DMM group, but this difference was not statistically significant. Remarkably, the combination of SBR and Pel-1.5 led to a substantial 1.6-fold increase in SOX9 expression compared to the SBR or Pel-1.5 groups alone. Notably, the expression intensity in the SBR + Pel group was similar to that of the Pel-4.5 group and closely resembled the SOX9 expression levels observed in the sham group (Figures 3F,G). Similarly, Col2a expression, a key structural protein in cartilage that provides strength and elasticity, was significantly decreased in the cartilage area induced by DMM. While there was a significant increase only in the SBR group, both the Pel-4.5 and SBR + Pel groups showed a significant increase compared to the DMM group. In particular, the SBR + Pel group achieved an expression level 1.5-fold higher than either treatment alone (Figures 3H,I). Aggrecan expression, one of the main components of cartilage, was significantly reduced by 3.8-fold in the DMM group compared to the sham group. The SBR and Pel-1.5 groups showed an average increase in aggrecan expression, but the SBR + Pel combination significantly increased expression by about 1.5-fold compared to the Pel-1.5 group alone (Figures 3J,K).

### 3.4 Synergistic inhibitory effects of combined SBR and pel therapy on cartilage-degrading enzymes and hypertrophic chondrocytes in OA

**FIGURE 4:**
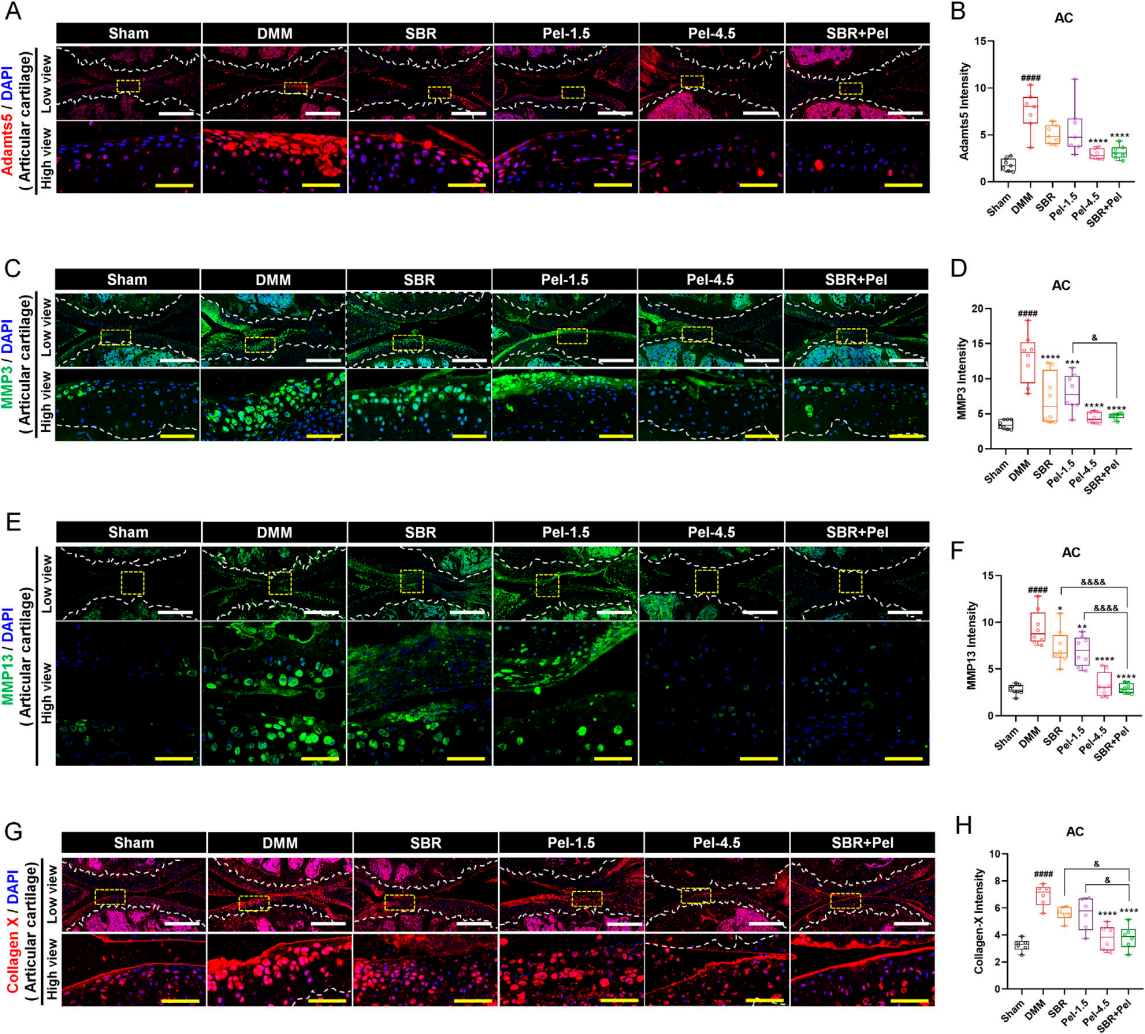
Immunohistological staining analysis of cartilage-degrading enzymes in joint tissues obtained at 8 weeks after DMM-induced OA, with SBR and Pel administrations. **(A,B)** Representative fluorescence images of Adamts5 (red) in cartilage of each group, along with a quantification graph measuring expression intensity. **(C,D)** Representative fluorescence images of MMP3 (green) in cartilage of each group, along with a quantification graph measuring the intensity of each marker. **(E,F)** Representative fluorescence images of MMP13 (green) in cartilage of each group, along with a quantification graph measuring expression intensity. **(G,H)** Representative fluorescence images of Collagen X (red) in cartilage of each group, along with a quantification graph measuring expression intensity. All white scale bar = 200 μm, all yellow scale bar = 50 μm. White dashed lines indicate the boundary of the articular cartilage, and yellow dashed squares in the low-magnification images mark the regions shown at higher magnification in the lower panels. DAPI (blue) was used as a nuclear counterstain in all fluorescence images. Abbreviations: AC, Articular Cartilage. Data are expressed as the mean ± standard deviation. Significant differences were analyzed via one-way analysis of variance with Tukey’s post-hoc analysis as follows: ^####^p < 0.0001 compared with Sham group; ^*^p < 0.05, ^**^p < 0.01, ^***^p < 0.001, and ^****^p < 0.0001 compared with DMM group; ^&^p < 0.05, ^&&^p < 0.01, and ^&&&&^p < 0.0001 compared with SBR or Pel-1.5 groups.

Upon performing IHC analysis for Adamts5, MMP3, MMP13, and collagen X, we found that Adamts5, an enzyme known to degrade proteoglycans in the cartilage, exhibited a significant 4-fold increase in expression following DMM induction (Figures 4A,B). Treatment with SBR or Pel-1.5 alone tended to reduce Adamts5 expression; however, the variation between mice prevented these changes from reaching statistical significance. In contrast, the Pel-4.5 and SBR + Pel groups exhibited a significant reduction in Adamts5 expression compared with the DMM group. Although the average reduction in expression was greater in the SBR + Pel group than in either the SBR or Pel-1.5 groups, the synergistic effect was not statistically significant. IHC analysis revealed that the expressions of MMP3 and MMP13, the enzymes responsible for collagen degradation in the cartilage, significantly 3-fold increased following DMM induction (Figures 4C–F). Both the SBR and Pel-1.5 monotherapy groups showed a significant reduction in MMP3 and MMP13 expressions compared with the DMM group. Notably, the SBR + Pel combination further decreased MMP3 and MMP13 expressions by approximately 2-fold compared to individual treatments, with effects similar to the Pel-4.5 group, rendering the enzyme expressions almost undetectable. Collagen X, a marker of hypertrophic chondrocytes, showed a significant 2-fold increase in expression after DMM induction (Figures 4G,H). SBR and Pel-1.5 treatments resulted in minimal reduction in collagen X expression, whereas Pel-4.5 and SBR + Pel treatments significantly decreased it by 1.8-fold compared to that in the DMM group. Additionally, the SBR + Pel group demonstrated a significant synergistic effect compared to the individual treatment groups.

### 3.5 Synergistic effects of SBR and pel combination therapy on cartilage formation and maintenance in OA-induced growth plate

**FIGURE 5:**
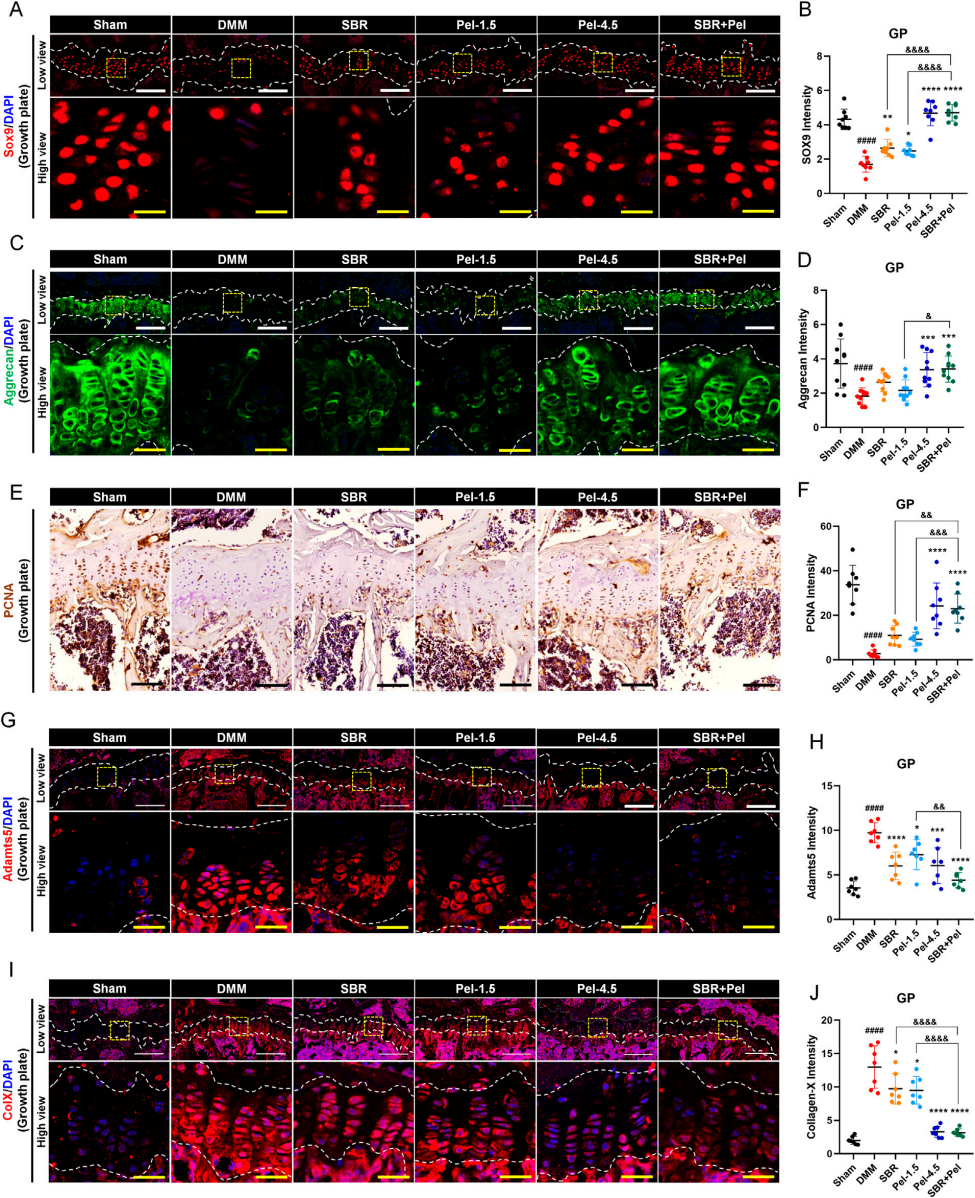
Immunohistological staining analysis evaluating synergistic effects of SBR and Pel combination therapy on cartilage integrity in the growth plate region of joint tissues obtained at 8 weeks after DMM-induced OA, with SBR and Pel administrations. **(A,B)** Representative fluorescence images of SOX9 (red) in growth plate in each group, along with a quantification graph measuring expression intensity. **(C,D)** Representative fluorescence images of aggrecan (green) in growth plate in each group, along with a quantification graph measuring the intensity of each marker. **(E,F)** Representative DAB images of PCAN in growth plate in each group, along with a quantification graph measuring expression intensity. Blank scale bar = 200 μm. **(G,H)** Representative fluorescence images of Adamnts5 (red) in growth plate in each group, along with a quantification graph measuring expression intensity. **(I,J)** Representative fluorescence images of Collagen X (red) in growth plate in each group, along with a quantification graph measuring expression intensity. All white scale bar = 200 μm, all yellow scale bar = 50 μm. White dashed lines indicate the boundary of the cartilage, and yellow dashed squares in the low-magnification images denote the regions shown at higher magnification in the lower panels. DAPI (blue) was used as a nuclear counterstain in all fluorescence images. Abbreviations: GP, Growth Plate. Data are expressed as the mean ± standard deviation. Significant differences were analyzed via one-way analysis of variance with Tukey’s post-hoc analysis as follows: ^####^p < 0.0001 compared with Sham group; ^*^p < 0.05, ^**^p < 0.01, ^***^p < 0.001, and ^****^p < 0.0001 compared with DMM group; ^&^p < 0.05, ^&&^p < 0.01, and ^&&&&^p < 0.0001 compared with SBR or Pel-1.5 groups.

To evaluate the impact of SBR + Pel combination therapy on cartilage formation and degradation, we compared the expression of various markers related to cartilage formation and degradation in the growth plate, an area crucial for cartilage regeneration and repair owing to its active chondrocyte proliferation and differentiation. This approach allowed us to more clearly assess the state of chondrocytes and the changes induced by the combination therapy. SOX9 expression level in the growth plate significantly decreased by 2.5-fold after DMM induction. However, SOX9 expression increased significantly in the SBR and Pel-1.5 groups compared to the DMM group, with the SBR + Pel group showing a much greater increase of 1.8-fold compared to the individual treatments. This significant increase in expression was comparable to the levels observed in the Pel-4.5 and sham groups (Figures 5A,B). Aggrecan expression, which significantly decreased in DMM-induced growth plates, showed no significant increase in the SBR and Pel-1.5 groups; however, it increased in the Pel-4.5 and SBR + Pel groups, with levels significantly higher in the latter than in the Pel-1.5 group (Figures 5C,D).

Additionally, to evaluate the proliferative capacity of chondrocytes in the growth plate, we conducted PCNA analysis. PCNA analysis revealed a significant decrease in PCNA expression after DMM induction. While the SBR and Pel-1.5 groups showed a trend toward increased expression, it was not significant. In contrast, the Pel-4.5 and SBR + Pel groups had significantly higher PCNA expression, with SBR + Pel showing a significant increase compared to individual treatments (Figures 5E,F). We also analyzed Adamts5 and collagen X, which are important markers in the pathological process of OA and are critical for evaluating cartilage degradation and cellular changes. After DMM induction, the expression levels of Adamts5 and collagen X significantly increased in the growth plate. Treatment with SBR or Pel-1.5 alone resulted in a significant reduction in their expression. Notably, the combination of SBR and Pel led to a significant decrease in these markers, with an average reduction greater than that observed in the Pel-4.5 group. However, statistically, the SBR + Pel group only showed a significant difference when compared with the Pel-1.5 group alone in the case of Adamts5 (Figures 5G–J).

### 3.6 Synergistic effects of combined SBR and pel therapy on safety and functional efficacy in mice with DMM-induced OA

**FIGURE 6:**
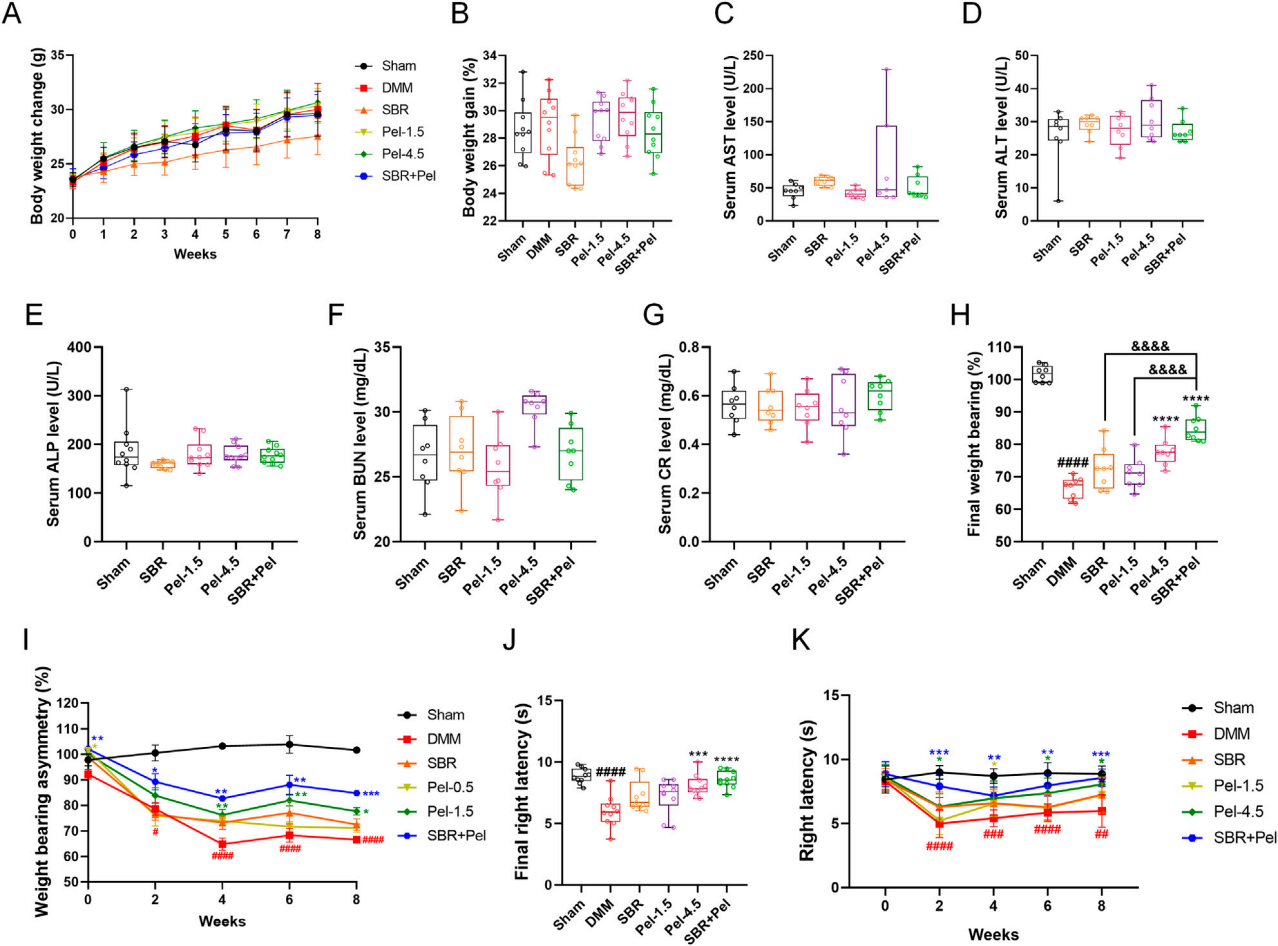
Evaluation of safety and efficacy of SBR and Pel combination therapy in a DMM-induced OA model **(A)** Body weight of each group measured weekly over the 8-week experimental period. **(B)** Percentage of body weight gain calculated as: body weight gain (%) = ((Final weight at 8 weeks–Initial weight at week 0)/Initial weight at week 0) × 100. **(C–E)** Blood chemistry tests for liver function indicators aspartate aminotransferase (AST), alanine aminotransferase (ALT), and alkaline phosphatase (ALP). Blood samples were collected from the retroorbital plexus under anesthesia at the 8-week sacrifice point. **(F,G)** Blood chemistry tests for kidney function indicators blood urea nitrogen (BUN) and creatinine (CR). **(H)** Final average values of weight-bearing capacity at week 8 for each group. **(I)** Weight-bearing asymmetry assessed every 2 weeks over the 8-week study period. **(J)** Final average latency (s) of right paw withdrawal measured by the von Frey test at week 8 for each group. **(K)** Right paw latency (s) measured every 2 weeks over the 8-week study period by the Von Frey test. Data are expressed as the mean ± standard deviation. Significant differences were analyzed via one-way analysis of variance with Tukey’s post-hoc analysis as follows: ^##^p < 0.01, ^###^p < 0.001, and ^####^p < 0.0001 compared with Sham group; ^*^p < 0.05, ^**^p < 0.01, ^***^p < 0.001, and ^****^p < 0.0001 compared with DMM group; ^&&&&^p < 0.0001 compared with SBR.

We evaluated safety by assessing body weight and liver and kidney function indicators and assessed the improvement in behavioral functions using weight-bearing and Von Frey tests. The SBR group showed a consistently lower average body weight, but there were no significant differences among the groups up to week 8 (Figure 6A). Analysis of the body weight gain percentage, calculated from the initial and final weights, showed a decreasing trend in the SBR group; however, this difference was not statistically significant across all groups (Figure 6B). Liver and kidney function indicators were analyzed in serum samples separated from the blood samples. For liver function, aspartate aminotransferase (AST) levels in the Pel-4.5 group were significantly higher (2/7 mice) than in the sham group; however, this was attributed to individual variations and did not result in statistical significance between the groups. The SBR, Pel-1.5, and SBR + Pel groups showed AST levels similar to those in the sham group, with no significant differences between the groups (Figure 6C). Other liver enzymes (ALT and ALP) and kidney function indicators (BUN and creatinine) showed no significant differences across all groups (Figures 6D–G).

We evaluated functional recovery in the OA model by assessing unilateral pain (specifically joint pain) using the classic method of measuring the weight distribution balance between both hind limbs, i.e., the weight-bearing test. The DMM group showed a significant decrease in weight-bearing asymmetry (%) compared with the sham group, with significant differences observed at each bi-weekly interval. In contrast, the Pel-4.5 and SBR + Pel groups demonstrated significant improvement compared with the DMM group, starting from the 2-week mark and continuing through the 8-week mark. Notably, at the final 8-week point, the SBR + Pel group showed significantly greater improvement than the SBR or Pel-1.5 alone groups (Figure 6H). The final rates of improvement in weight-bearing, calculated relative to the DMM-induced OA control group, revealed that the SBR group had an improvement rate of 8.9%, and the Pel-1.5 group had a rate of 6.9%. In contrast, the Pel-4.5 and SBR + Pel groups showed significantly higher improvement rates (16.7% and 27.4%, respectively; Figure 6I). These results indicated that single treatments with SBR or Pel-1.5 resulted in minimal improvement rates of <10%, demonstrating limited efficacy when administered alone. Conversely, the combined treatment with 100-mg/kg SBR and 0.5-mg/kg Pel exhibited an outstanding improvement rate exceeding 27%, highlighting the superior efficacy of combination therapy in enhancing weight-bearing capacity.

Additionally, we conducted the Von Frey test to assess the differences in pain sensitivity between the groups after mechanical stimulation. There was a significant decrease in latency in the DMM group compared with the sham group. Although the latency showed an increasing trend over the 8-week period, significant differences remained when compared with the sham group. The SBR + Pel group showed a significant increase in latency starting from the 2-week mark compared with the DMM group. Specifically, at the final 8-week point, the Pel-4.5 and SBR + Pel groups exhibited significant differences compared with the DMM group. However, no significant differences were observed when compared to the SBR or Pel-1.5 alone groups (Figure 6J). Over the 8-week evaluation period, the sham group consistently maintained significantly higher latency values compared with the DMM group, with mean values ranging from 8.44 to 8.99 s. Notably, the combination treatment of SBR and Pel-1.5 resulted in an improvement rate of 43.4%, which was markedly greater than that observed with SBR (15.3%) or Pel-1.5 (7.8%) administered alone (Figure 6K).

## 4 Discussion

This study provides a strong foundation for the development of novel combination therapies for OA to achieve enhanced therapeutic outcomes. These findings provide compelling evidence that SBR + Pel offers enhanced therapeutic benefits compared with individual treatments, with a favorable safety profile. Our results also demonstrated that SBR + Pel significantly improved cartilage regeneration and maintenance in both *in vitro* and *in vivo* models. SBR is a traditional herbal medicine composed of ingredients such as Gucheok, Bangpung, Useul, Ohgapi, Duchung, and Heukdu. It is known to exert anti-inflammatory and chondroprotective effects by modulating the production of inflammatory cytokines (TNF-α, IL-1β) and enzymes (COX-2, iNOS, MMPs) within joint tissues while reducing oxidative stress (Jung et al., 2019; Kim et al., 2016). In contrast, Pel is an NSAID that reduces prostaglandin synthesis by inhibiting COX enzymes. Clinical trials indicate it to be as effective as celecoxib, a globally used COX-2 inhibitor, in controlling pain associated with rheumatoid arthritis (Choi et al., 2014; Shin et al., 2011). However, long-term use of Pel can increase the risk of severe gastrointestinal side effects, and, similar to other NSAIDs, high doses may increase the risk of chronic renal failure (Baker and Perazella, 2020; Drozdzal et al., 2021).

In this preclinical study evaluating the effectiveness and safety of combination therapy using Pel and the natural OA treatment SBR, the CCK-8 analysis identified optimal concentrations of 200 μg/mL for SBR and 25 μM for Pel, and this optimal combination showed synergistic effects on reducing cytotoxicity, inhibiting NO production, and preventing chondrocyte degeneration. For *in vivo* experiments, the dose selection was based on previously published literature, which reported that SBR exhibits anti-inflammatory effects starting at 100 mg/kg and Pel exerts anti-inflammatory and analgesic effects from 0.5 mg/kg (Kim et al., 2011; Yoon et al., 2023). Considering these findings and the potential for synergistic interaction, we selected SBR at 100 mg/kg and Pel at 1.5 mg/kg to achieve maximal efficacy at minimal dosage. In addition, a higher Pel dose of 4.5 mg/kg was applied, as it represents the next concentration reported in the literature following 1.5 mg/kg. In the DMM-induced OA model, the combination therapy with 100 mg/kg SBR and 1.5 mg/kg Pel improved cartilage integrity, reduced synovitis scores, and synergistically increased collagen content. Immunohistochemical analysis also revealed synergistic effects in upregulating cartilage markers (SOX9, Col2a1, aggrecan) and inhibiting cartilage-degrading enzymes (Adamts5, MMP3, MMP13). Furthermore, the reduction in collagen X expression suggests that this combination therapy may slow the progression of OA. We also analyzed the expression of these markers in the growth plate and found that similar to the results observed in the cartilage, SBR + Pel demonstrated synergistic effects in the OA-induced growth plate. This consistency in results across different tissues highlights the robust efficacy of SBR + Pel in promoting cartilage health and preventing OA progression. Previous research has shown that combining natural substances and pharmaceuticals can potentially reduce the required dose of each ingredient, lower the risk of side effects, and achieve more effective treatment by targeting multiple processes involved in the disease (Izzo, 2005; Rombola et al., 2020). SBR enhances the anti-inflammatory response by modulating various pathways, whereas Pel selectively inhibits COX-2 to reduce prostaglandin synthesis. The synergistic effects of SBR and Pel likely stem from complementary mechanisms, although the specific pathways and interactions contributing to their combined efficacy remain unclear. The safety evaluation showed that liver enzymes and kidney function indicators remained within normal ranges, indicating no significant adverse effects on liver and kidney function or body weight. Although there was no statistical significance, two mice in the Pel-4.5 group exhibited elevated serum AST levels. Therefore, the combination of SBR + Pel suggests that it may provide an effective treatment option with a reduced risk of side effects compared to high-dose Pel therapy alone.

Despite these promising results, this study did not fully elucidate the complementary mechanisms of action underlying the synergistic effects of SBR and Pel. In particular, potential pharmacokinetic interactions between the two agents such as their effects on drug metabolism, distribution, and excretion were not assessed. These interactions may influence the overall efficacy and safety profile of the combination therapy, highlighting the need for further pharmacokinetic and pharmacodynamic investigations. Moreover, this study did not include comprehensive *in vivo* safety assessments, such as histopathological analysis of major organs, long-term toxicity studies, or evaluation of potential immune responses. Future research should therefore address these limitations in greater detail and incorporate translational studies using human cell lines and clinical trials to confirm the efficacy and safety of SBR + Pel in patients with OA. A more thorough understanding of both pharmacological mechanisms and safety interactions will be essential for optimizing this combination therapy and advancing OA treatment.

### 4.1 Conclusion

SBR + Pel offers a promising and safer treatment strategy for OA compared to individual therapies. The observed synergistic effects on cartilage regeneration, inflammation reduction, and functional recovery underscore its therapeutic potential. Future research should analyze the molecular mechanisms behind these effects and evaluate long-term efficacy and safety in human cell lines and clinical trials to support clinical application.

## Data Availability

The original contributions presented in the study are included in the article/supplementary material, further inquiries can be directed to the corresponding author.
